# Effectiveness of vaccination against SARS-CoV-2 infection and Covid-19 hospitalisation among Finnish elderly and chronically ill—An interim analysis of a nationwide cohort study

**DOI:** 10.1371/journal.pone.0258704

**Published:** 2021-11-18

**Authors:** Ulrike Baum, Eero Poukka, Arto A. Palmu, Heini Salo, Toni O. Lehtonen, Tuija Leino

**Affiliations:** 1 Infectious Disease Control and Vaccinations Unit, Department of Health Security, Finnish Institute for Health and Welfare (THL), Helsinki, Finland; 2 Population Health Unit, Department of Public Health and Welfare, Finnish Institute for Health and Welfare (THL), Tampere, Finland; Mahidol Oxford Clinical Research Unitl (MORU), THAILAND

## Abstract

**Background:**

In Finland, both mRNA and adenovirus vector (AdV) Covid-19 vaccines have been used after the vaccination campaign started on December 27, 2020. Vaccination of the elderly and chronically ill was prioritized and the interval between doses set to 12 weeks. The objective of this interim analysis was to evaluate first and second dose vaccine effectiveness (VE) in a real-world setting.

**Methods:**

During the first five months of the campaign, a register-based cohort study was conducted in the Finnish elderly aged 70+ years and those aged 16–69 years with medical conditions predisposing to severe Covid-19 (chronically ill). Using Cox regression, VE against SARS-CoV-2 infection and Covid-19 hospitalisation was estimated comparing the hazard in the vaccinated with that in the unvaccinated.

**Results:**

The cohorts included 901092 elderly (89% vaccinated) and 774526 chronically ill (69% vaccinated) individuals. Three weeks after the first dose, mRNA VE against infection was 45% (95% confidence interval, 36–53%) and 40% (26–51%) in elderly and chronically ill; mRNA VE against hospitalisation was 63% (49–74%) and 82% (56–93%). In chronically ill, AdV VE was 42% (32–50) and 62% (42–75%) against infection and hospitalisation, respectively. One week after the second dose, mRNA VE against infection was 75% (65–82%) and 77% (65–85%) in elderly and chronically ill; mRNA VE against hospitalisation was 93% (70–98%) and 90% (29–99%).

**Conclusions:**

Covid-19 vaccines protect against SARS-CoV-2 infection and Covid-19 hospitalisation. A single dose provides moderate protection in elderly and chronically ill, although two doses are clearly superior.

## Introduction

The global burden of the ongoing Covid-19 pandemic caused by the severe acute respiratory syndrome coronavirus 2 (SARS-CoV-2) has been immense. Finland, a Nordic country with 5.5 million inhabitants, has been less affected than the rest of Europe [1]. As of 24 May 2021, there have been roughly 90 000 confirmed Covid-19 cases and 942 Covid-19-associated deaths in the Finnish population [2].

In late 2020 the European Medicines Agency authorized the first Covid-19 vaccines for use in the European Union [3]. Finland started its vaccination campaign promptly after, on 27 December prioritizing healthcare workers treating Covid-19 patients as well as residents and healthcare workers of long-term care facilities [4]. Thereafter, the elderly aged 70+ years and individuals aged 16+ years with (highly) predisposing medical conditions for severe Covid-19 were vaccinated [4, 5]. Due to vaccine shortage, Finland decided to postpone the second dose until 12 weeks after the first, similarly as in the United Kingdom (UK) [6], to provide more people with at least one dose as early as possible. Therefore, most of the elderly and chronically ill were vaccinated only once in the first quarter of 2021 [7]. By 24 May, two messenger ribonucleic acid (mRNA) vaccines (by BioNTech/Pfizer and Moderna) and two adenovirus vector (AdV) vaccines (by Oxford/AstraZeneca and Janssen) have been approved by the European Medicines Agency [8] but only the first three have so far been used in Finland.

The first cohort studies assessing the effectiveness of Covid-19 vaccines were published in early 2021. The vaccine effectiveness (VE) estimates for two doses from BioNTech/Pfizer varied between 86% in healthcare workers and 92% in nationwide settings and, thus, were close to the vaccine efficacy measured in randomized controlled trials [9, 10]. However, VE in the elderly and immunocompromised individuals has been suspected to be lower [11].

The aim of this observational study was to evaluate the interim effectiveness of Covid-19 vaccines in two cohorts, i.e. the elderly and the chronically ill, during the first five months of the vaccination campaign in Finland. As the administration of the second dose has been postponed, we focus especially on how the VE is sustained after the first dose.

## Methods

To estimate effectiveness of Covid-19 vaccines in the elderly and the chronically ill in Finland, we conducted a nationwide cohort study based on data from eight national registers linked using the unique Finnish personal identity code. The study follow-up begun with the first Covid-19 vaccinations in Finland (27 December 2020) and ended on 24 May 2021. The target populations were all the elderly aged 70+ years and all the chronically ill aged 16–69 years at the beginning of the study. All study subjects were permanent residents and registered in the Population Information System, which provided information on each individual’s date of birth and death (if applicable), sex and municipality of residence.

The cohort of the chronically ill was formed by individuals identified to be at increased risk of severe Covid-19 due to certain medical conditions. These conditions included active cancer treatment, organ or stem cell transplant, severe disorders of the immune system, severe chronic renal, liver or pulmonary disease, type 1 and type 2 diabetes mellitus, Down syndrome, severe heart disease, neurological illness or condition affecting breathing, immunosuppressive drug therapy for autoimmune disease, adrenal gland disorders, sleep apnea and psychotic disorders (S1 Table).

Distinguishing between predisposing and highly predisposing conditions (S1 Table), presence of the aforementioned medical conditions was determined in both cohorts using the diagnostic data in the Care Register for Health Care and the Register of Primary Health Care Visits since 2015 as well as the data in the Special Reimbursement Register and Prescription Centre database maintained by the Social Insurance Institution. The Special Reimbursement Register allowed the identification of all individuals entitled, at least until 1 January 2020, to a special reimbursement for medical expenses, indicating that the entitled individual met explicit criteria for selected medical conditions (S1 Table). The Prescription Centre database was used to identify all individuals using selected medications of interest (S1 Table).

The outcomes of interest were SARS-CoV-2 infection, laboratory-confirmed through polymerase chain reaction or antigen test as registered in the National Infectious Diseases Register, and Covid-19 hospitalisation, recorded in the Care Register for Health Care, timely associated with a confirmed SARS-CoV-2 infection. Covid-19 hospitalisation was defined as any inpatient hospital admission with Covid-19 (International Classification of Diseases, tenth revision, codes: U07.1, U07.2), acute respiratory tract infections (J00–J22, J46) or severe complications of lower respiratory tract infections (J80–84, J85.1, J86) as primary or secondary diagnosis. Hospital admissions and SARS-CoV-2 infections were timely associated if the infection was confirmed up to 14 days before or 7 days after the admission. Individuals with a confirmed SARS-CoV-2 infection prior to the study follow-up (total number less than 1% of target population) were excluded from the cohorts.

The exposure of interest was vaccination against Covid-19 with at least one dose of any vaccine, exactly one or two doses of mRNA vaccine, or exactly one or two doses of AdV vaccine (chronically ill only). The vaccine and date of vaccination were extracted from the National Vaccination Register. The time since vaccination with the first dose was split into the intervals 0–6 days since vaccination (DSV), 7–13 DSV, 14–20 DSV, 21–27 DSV, 28–34 DSV, 35–41 DSV and 42+ DSV. The time since vaccination with the second dose was split into the intervals 0–6 DSV and 7+ DSV. All study subjects entered their cohort unvaccinated. When they were then vaccinated during the follow-up, their exposure status changed over time.

Each study subject was considered to be at risk of the outcome of interest (confirmed SARS-CoV-2 infection or Covid-19 hospitalisation) from the beginning of the study until the first occurrence of any of the following events: outcome of interest, death, end of study, or day 14 after confirmed SARS-CoV-2 infection (if the outcome of interest was Covid-19 hospitalisation). Using Cox regression with time in study as the underlying time scale, we compared the hazard of confirmed SARS-CoV-2 infection or Covid-19 hospitalisation in vaccinated study subjects with the hazard in the unvaccinated. The effect measure of interest was VE, quantified as one minus the hazard ratio adjusted for age group (chronically ill: 16–38, 39–51, 52–59, 60–64, 65–69 years (age distribution quintiles); elderly: 70–74, 75–79, 80–89, 90+ years), sex, presence of medical conditions predisposing to severe Covid-19 (S1–S3 Tables and Tables 1–3), residence in the most affected hospital district Helsinki-Uusimaa (1.7 million inhabitants), and residence in long-term care (elderly only).

**Table 1:** mRNA vaccine effectiveness in the Finnish elderly aged 70+ years adjusted for age group, sex, presence of medical conditions, residence in Helsinki-Uusimaa hospital district and residence in long-term care.

	Laboratory-confirmed SARS-CoV-2 infection			Covid-19 hospitalisation		
	Cases, n	Cumulative risk^a^	Vaccine effectiveness^b^, %	Cases, n	Cumulative risk^a^	Vaccine effectiveness^b^, %
Not vaccinated	1526	295	Reference	319	73	Reference
**First dose**						
21–27 DSV	71	349	41 (25–54)	14	33	57 (24–75)
28–34 DSV	59	135	47 (30–59)	13	28	59 (26–77)
35–41 DSV	56	233	46 (28–59)	11	57	64 (32–81)
42+ DSV	197	104	46 (35–56)	39	12	68 (50–79)
**Second dose**						
7+ DSV	45	132	75 (65–82)	2	3	93 (70–98)

^a^ Cumulative risk at the end of the study multiplied by 10000;

^b^ Point estimate and 95% confidence interval; DSV, days since vaccination.

**Table 2:** mRNA vaccine effectiveness in the chronically ill aged 16–69 years adjusted for age group, sex, presence of medical conditions and residence in Helsinki-Uusimaa hospital district.

	Laboratory-confirmed SARS-CoV-2 infection			Covid-19 hospitalisation		
	Cases, n	Cumulative risk^a^	Vaccine effectiveness^b^, %	Cases, n	Cumulative risk^a^	Vaccine effectiveness^b^, %
Not vaccinated	5280	859	Reference	528	90	Reference
**First dose**						
21–27 DSV	35	242	41 (17–58)	1	2	89 (24–99)
28–34 DSV	20	217	58 (34–73)	1	2	86 (-1 to 98)
35–41 DSV	13	294	59 (29–76)	0	0	Not estimated
42+ DSV	43	431	14 (-16 to 37)	3	13	59 (-29 to 87)
**Second dose**						
7+ DSV	20	181	77 (65–85)	1	32	90 (29–99)

^a^ Cumulative risk at the end of the study multiplied by 10000;

^b^ Point estimate and 95% confidence interval; DSV, days since vaccination.

**Table 3:** Adenovirus vector vaccine effectiveness in the chronically ill aged 16–69 years adjusted for age group, sex, presence of medical conditions and residence in Helsinki-Uusimaa.

	Laboratory-confirmed SARS-CoV-2 infection			Covid-19 hospitalisation		
	Cases, n	Cumulative risk^a^	Vaccine effectivenes, %	Cases, n	Cumulative risk^a^	Vaccine effectiveness^b^, %
Not vaccinated	5280	859	Reference	528	90	Reference
**First dose**						
21–27 DSV	51	238	24 (-1 to 42)	4	42	67 (10–88)
28–34 DSV	29	129	48 (25–64)	1	3	91 (33–99)
35–41 DSV	29	184	37 (8–56)	6	37	36 (-45 to 72)
42+ DSV	78	106	50 (37–61)	15	21	58 (26–76)
**Second dose**						
7+ DSV	0	0	Not estimated	0	0	Not estimated

^a^ Cumulative risk at the end of the study multiplied by 10000;

^b^ Point estimate and 95% confidence interval; DSV, days since vaccination.

All analyses were performed in R 3.6.3 (R Foundation for Statistical Computing, Vienna, Austria).

### Ethical issues and funding statement

The study was conducted under the Finnish Communicable Disease Act and is part of the Finnish Institute for Health and Welfare surveillance duty to monitor the efficacy of vaccines [12]. The Finnish Institute for Health and Welfare has legal right to use registers in the surveillance duty and, therefore, the study did not require further ethical review. All data were pseudonymised prior to access.

Because the study was part of the Finnish Institute for Health and Welfare surveillance duty, no external funding was used.

## Results

**Fig 1:**
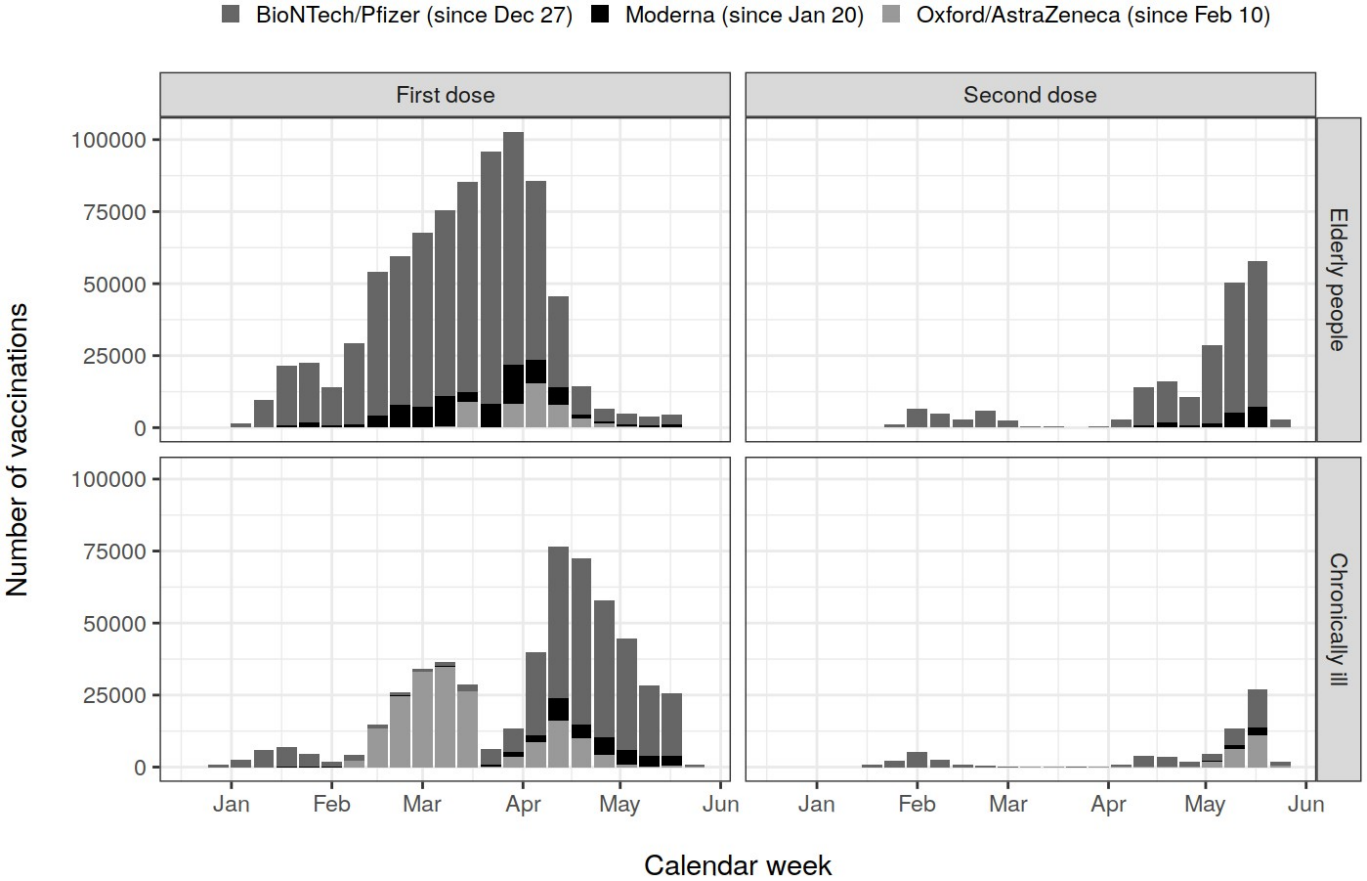
Number of vaccinations administered in the Finnish elderly aged 70+ years and chronically ill aged 16–69 years by vaccine manufacturer and calendar week.

The study cohorts of the elderly and the chronically ill included 901 092 and 774 526 individuals, respectively, of which 89% and 69% were vaccinated at least once against Covid-19 during the study follow-up. More than 90% of the vaccinated elderly had received mRNA vaccine, while among the chronically ill two thirds of the vaccinees had received mRNA vaccine; the rest had received AdV vaccine from Oxford/AstraZeneca (Fig 1). Only a small proportion was vaccinated twice (Fig 1). In the elderly, the vaccination coverage was similar across age groups, sex, and hospital districts (S2 Table). In the chronically ill, the vaccination coverage increased with age (S3 Table).

**Fig 2:**
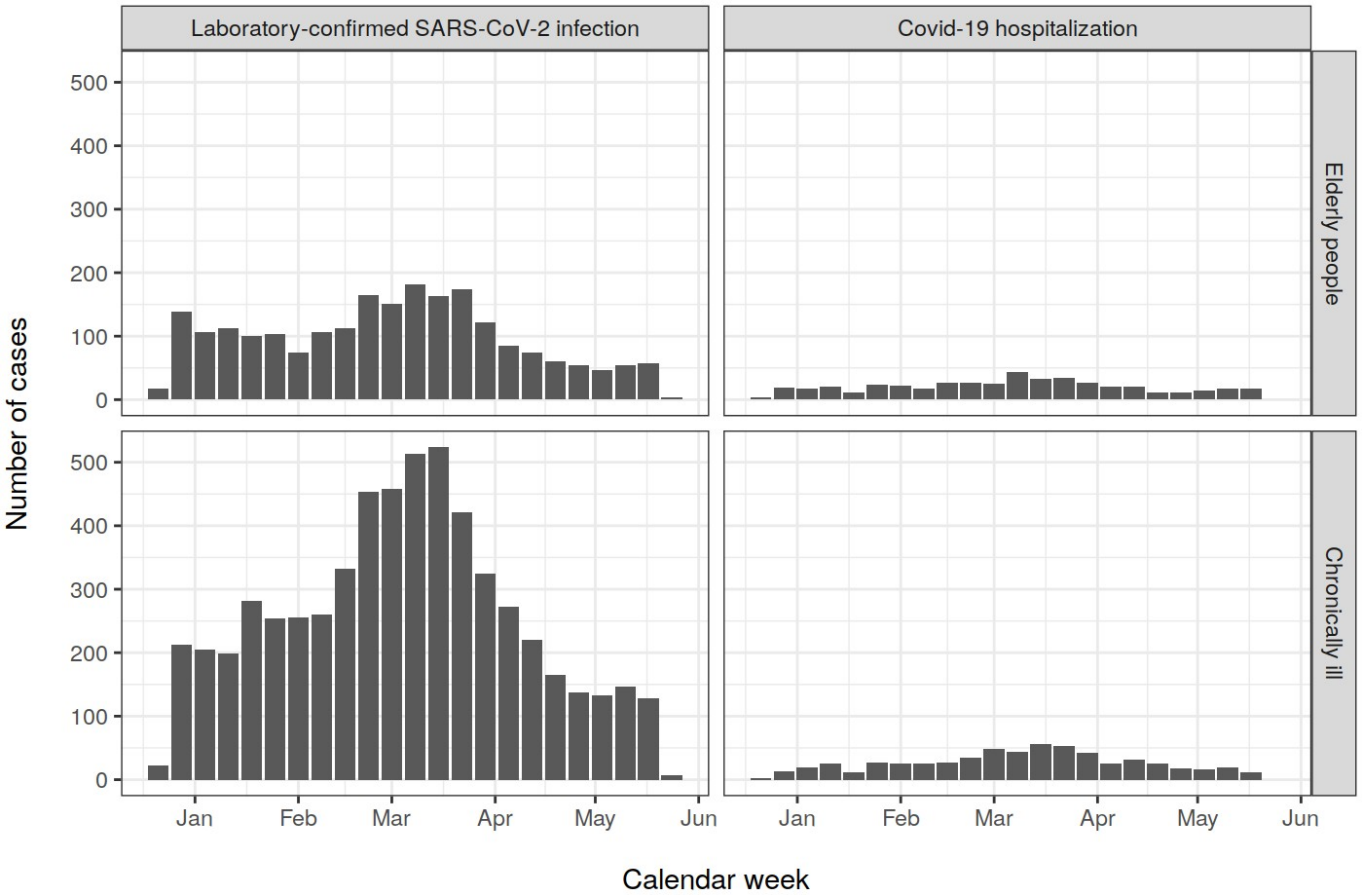
Number of cases of confirmed SARS-CoV-2 infection and Covid-19 hospitalisation in the Finnish elderly aged 70+ years and chronically ill aged 16–69 years by calendar week.

In total, there were 2265 cases of confirmed SARS-CoV-2 infection and 466 cases of Covid-19 hospitalisation among the elderly and 5929 cases of confirmed infection and 598 cases of hospitalisation among the chronically ill (Fig 2). The VE against confirmed infection 21+ days since the first vaccination with any vaccine was 51% (95% confidence interval: 44–58%) in the elderly and 48% (41–54%) in the chronically ill. The corresponding VE against hospitalisation was 66% (53–76%) and 69% (55–79%), respectively.

In both cohorts, the hazards of confirmed infection and hospitalisation during the follow-up of less than seven days since first vaccination were significantly lower than the corresponding hazards in the unvaccinated follow-up (S4–S6 Tables). This initial difference between the vaccinated and unvaccinated decreased during the first 2–3 weeks. VE estimates against confirmed infection and hospitalisation started to rise 14 and 21 days after vaccination.

In the elderly, the effectiveness of exactly one dose of any mRNA vaccine against confirmed infection and hospitalisation 21+ DSV was 45% (95% confidence interval: 36–53%) and 63% (49–74%) respectively. After the first three weeks since vaccination, VE against infection was constant, while VE against hospitalisation further increased over time (Table 1). After the second mRNA vaccination, VE against confirmed infection and hospitalisation was 75% (65–82%) and 93% (70–98%) (Table 1).

In the chronically ill, the effectiveness of exactly one dose of any mRNA vaccine against confirmed infection and hospitalisation 21+ DSV was 40% (95% confidence interval: 26–51%) and 82% (56–93%), respectively. Although initially constant, VE appeared to decrease after 42 days (Table 2). However, the confidence intervals are broad and overlapping. After the second mRNA vaccination, VE against confirmed infection and hospitalisation was 77% (65–85%) and 90% (29–99%) (Table 2).

The effectiveness of exactly one dose of AdV vaccine against confirmed infection and hospitalisation 21+ DSV was 42% (95% confidence interval: 32–50%) and 62% (42–75%), respectively. The VE estimates fluctuated over time but did not decrease after 42 days (Table 3). Due to the small number of vaccinees given two doses of AdV vaccine (Fig 1), the effectiveness of the second AdV vaccination could not be estimated (Table 3).

## Discussion

The present observational, population-based study quantified the interim effectiveness of Covid-19 vaccines in the elderly and the chronically ill during the first five months of the vaccination campaign in Finland. Three weeks after the first dose, VE was moderate against confirmed SARS-CoV-2 infection but considerably higher against Covid-19 hospitalisation. After the first mRNA vaccination (BioNTech/Pfizer or Moderna), the VE estimates were constant in the elderly from the fourth through the seventh week and beyond. However, we were not able to demonstrate sustained mRNA VE in the chronically ill for all post-vaccination intervals. Nevertheless, the second mRNA vaccination increased the VE estimates substantially, both in the elderly and the chronically ill. After the first AdV vaccination (Oxford/AstraZeneca), the observed VE sustained through all post-vaccination intervals in the study but VE of the second dose could not be estimated.

Several studies have previously evaluated VE against SARS-CoV-2 infection. An observational, population-based study from Israel estimated VE among the elderly aged 70+ years at 50 and 95% three weeks after the first dose and one week after the second dose of the BioNTech/Pfizer vaccine, respectively [9]. Although similar, these results might, however, not be fully comparable to our results as all individuals confined to their homes or long-term care facilities were excluded from the Israeli study but included in ours. In another study from Israel, VE against asymptomatic and symptomatic infection was 52% and 63%, respectively, in the third week after the first dose [13]. In England, VE against symptomatic infection in elderly aged 70+ years was estimated at 50% in the fifth week after the first dose from BioNTech/Pfizer and 61% after the first dose from Oxford/AstraZeneca [14]. In agreement with our findings, the results by Lopez Bernal et al. [14] thus indicate that VE levels sustain even beyond the first three weeks after the first dose.

Previous studies have also assessed VE against more severe outcomes and, in line with our study, detected higher protection levels against Covid-19 hospitalisation than infection. In the third week after the first dose from BioNTech/Pfizer, VE against hospitalisation was found to be 74% and 76% in the general Israeli population aged 16+ years [9, 13]. In the subsequent weeks VE of the first BioNTech/Pfizer dose ranged between 77 and 91% according to a Scottish cohort study [15]. When stratified by age, the VE after seven weeks was 76% among those aged 65–79 years and 85% among those aged 80+ years [15]. While the results of those studies are similar, our findings suggest that the VE of only one dose might be lower. Nevertheless, the present estimates concerning the VE of two doses are comparable to the previously published figures ranging between 87 and 97% [9, 13, 16].

Although a direct comparison of the effectiveness estimates for mRNA and AdV vaccines may be influenced by the different dates the vaccines were introduced in the population (Fig 1), we did not detect meaningful differences. This finding is in line with other studies that analyzed the effectiveness of both mRNA and AdV vaccines against SARS-CoV-2 infection [14] and Covid-19 hospitalisation [14, 15, 17].

Although our VE estimates were roughly similar to those obtained in previous studies [9, 13–15], our estimates appear to be at the lower side of the spectrum. One reason might be that in Finland vaccinations were given first to the oldest among the elderly and the most immunocompromised among the chronically ill. High age seems to affect the effectiveness of Covid-19 vaccines less than expected based on the experience with other vaccines [9, 11, 13, 15]. Nevertheless, VE was found to be lower among the very old, the immunocompromised and residents of long-term care facilities [11, 18] and to vary across different predisposing medical conditions [11, 19].

In Finland, Alpha was the predominant SARS-CoV-2 variant causing 60–70% of all SARS-CoV-2 infections in the capital region and 50% of all SARS-CoV-2 infections in the rest of the country in March 2021. At the same time, Beta caused only 5% of the infections [20], but local reports indicate that the incidence of Beta has increased. Recently, there has been a concern that certain SARS-CoV-2 variants are associated with higher transmissibility [21], mortality [22], antibody resistance [23] and capability to cause Covid-19 epidemics in a population with high seroprevalence [24]. It has been shown that Covid-19 vaccines are effective against Alpha [13, 25, 26] but their efficacy against Beta seems to be reduced [27]. VE in Finland might, therefore, be decreased due to the increased incidence of Beta.

The main strengths of this analysis are the size and representativeness of the two cohorts and the register-based nature of the study [28]. In Finland, the recording of Covid-19 vaccinations into the patient information system is mandatory, as is the notification of SARS-CoV-2 positive laboratory findings. These data are then automatically transferred into the national registers. Because exposure, outcome and covariate data are recorded directly at the source and independently, the chance of measurement and recall bias has been minimized. By using Cox regression with time in study as the underlying time scale, we controlled any temporal changes in the hazard of infection and disease or detection of those. Moreover, it can be assumed that a great proportion of symptomatic infections have been captured since the threshold for testing was very low and testing capacities were at no point exhausted. Covid-19 testing has been free of charge and its importance emphasized through official channels.

A limitation of this study is the unknown proportion of asymptomatic cases among the confirmed infections. As VE is greater when measured against a more severe outcome [9], the proportion of asymptomatic cases strongly influences the VE estimates. The major weakness is, however, the presence of time-limited selection bias as in Lopez Bernal et al. [14]. At the day of vaccination, vaccinees were healthier than average due to the policy to postpone the vaccination of individuals with acute symptoms suggestive of Covid-19 and those in self-isolation. Consequently, the hazards of infection and hospitalisation in the recently vaccinated were lower than the hazards in the unvaccinated. In the absence of any bias, these hazards should have been similar in the vaccinated and unvaccinated as it takes 2–3 weeks for the vaccine-induced immune response to develop. In addition, residual confounding may be present due to the observational nature of the study.

This interim analysis covered only the early phase of the Covid-19 vaccination campaign in Finland. Future analyses should therefore also target less frequent, more severe outcomes, such as death or admission to an intensive care unit, and subpopulations who became eligible for vaccination later in 2021. In addition, an extension of the follow-up should allow more elaborate analyses of the waning of vaccine-induced immunity over time, the impact of SARS-CoV-2 variants on the VE, and differences in the effectiveness of different Covid-19 vaccine brands.

## Conclusions

Covid-19 vaccines protect against SARS-CoV-2 infection and Covid-19 hospitalisation. We detected no difference between mRNA and AdV vaccines. In the elderly and the chronically ill, a single dose provides moderate protection even beyond the first three weeks since vaccination, yet the full series of two doses is superior. Further studies are needed to assess Covid-19 vaccine effectiveness for different brands, schedules and target groups.

## Supporting information

S1 Table Definition of medical conditions (highly) predisposing to severe Covid-19. Registers: 1, Special Reimbursement Register for Medicine Expenses; 2, Care Register for Health Definition of medical conditions (highly) predisposing to severe Covid-19. ATC, Anatomical Therapeutic Chemical Classification System; ICD-10, International Statistical Classification of Diseases and Related Health Problems, tenth revision; ICPC-2, International Classification of Primary Care, second edition; NCSP, Nordic Nomesco Classification of Surgical Procedures. Registers: 1, Special Reimbursement Register for Medicine Expenses; 2, Care Register for Health Care; 3, Register of Primary Health Care Visits; 4, Prescription Centre database. ATC, Anatomical Therapeutic Chemical Classification System; ICD-10, International Statistical Classification of Diseases and Related Health Problems, tenth revision; ICPC-2, International Classification of Primary Care, second edition; NCSP, Nordic Nomesco Classification of Surgical Procedures. Registers: 1, Special Reimbursement Register for Medicine Expenses; 2, Care Register for Health Care; 3, Register of Primary Health Care Visits; 4, Prescription Centre database.
(PDF)

S2 Table Distribution of baseline characteristics and percentage vaccinated first with mRNA or adenovirus vector (AdV) vaccine, Finnish elderly aged 70+ years. (PDF)

S3 Table Distribution of baseline characteristics and percentage vaccinated first with mRNA or adenovirus vector (AdV) vaccine, chronically ill aged 16–69 years. (PDF)

S4 Table Crude and adjusted hazard ratios comparing the hazard of confirmed SARS-CoV-2 infection or Covid-19 hospitalization in study subjects who received exactly 1 or 2 doses of mRNA vaccine with the corresponding hazard in the unvaccinated, Finnish elderly aged 70+ years. DSV, day since vaccination; Est., Point estimate; LCI, lower 95% confidence interval limit; UCI, upper 95% confidence interval limit.
(PDF)

S5 Table Crude and adjusted hazard ratios comparing the hazard of confirmed SARS-CoV-2 infection or Covid-19 hospitalization in study subjects who received exactly 1 or 2 doses of mRNA vaccine with the corresponding hazard in the unvaccinated, chronically ill aged 16–69 years. DSV, day since vaccination; Est., Point estimate; LCI, lower 95% confidence interval limit; UCI, upper 95% confidence interval limit.
(PDF)

S6 Table Crude and adjusted hazard ratios comparing the hazard of confirmed SARS-CoV-2 infection or Covid-19 hospitalization in study subjects who received exactly 1 or 2 doses of adenovirus vector vaccine with the corresponding hazard in the unvaccinated, chronically ill aged 16–69 years. DSV, days since vaccination; Est., Point estimate; LCI, lower 95% confidence interval limit; UCI, upper 95% confidence interval limit.
(PDF)
