# Supplementary material for: Effectiveness of vaccination against SARS-CoV-2 infection and Covid-19 hospitalisation among Finnish elderly and chronically ill—An interim analysis of a nationwide cohort study
Source: PLoS One. 2021 Nov 18;16(11):e0258704. doi: 10.1371/journal.pone.0258704 (PMC8601574; doi:10.1371/journal.pone.0258704)
Supplement: S1 Table — Registers: 1, Special Reimbursement Register for Medicine Expenses; 2, Care Register for Health Definition of medical conditions (highly) predisposing to severe Covid-19. ATC, Anatomical Therapeutic Chemical Classification System; ICD-10, International Statistical Classification of Diseases and Related Health Problems, tenth revision; ICPC-2, International Classification of Primary Care, second edition; NCSP, Nordic Nomesco Classification of Surgical Procedures. Registers: 1, Special Reimbursement Register for Medicine Expenses; 2, Care Register for Health Care; 3, Register of Primary Health Care Visits; 4, Prescription Centre database. ATC, Anatomical Therapeutic Chemical Classification System; ICD-10, International Statistical Classification of Diseases and Related Health Problems, tenth revision; ICPC-2, International Classification of Primary Care, second edition; NCSP, Nordic Nomesco Classification of Surgical Procedures. Registers: 1, Special Reimbursement Register for Medicine Expenses; 2, Care Register for Health Care; 3, Register of Primary Health Care Visits; 4, Prescription Centre database. (PDF) [file pone.0258704.s001.pdf]

S1 Table: Definition of medical conditions (highly) predisposing to severe Covid-19.

Medical condition	Classification	Codes	Register
**Highly predisposing to severe Covid-19**			
Organ or stem cell transplant	ICD-10	T86, Z94	1,2
Active cancer treatment	ICD-10	C00–C97 (except C44), D05.1, D39	1,2
Severe disorders of the immune system	ICD-10	D70.8, D80–D84, E31.00	2
Severe chronic renal disease	ICD-10	I12, I13, N00–N05, N07, N08, N11, N14, N18, N19, E10.2, E11.2, E14.2	1,2
Asthma requiring continuous medication	ICD-10	J45, J46	2, 3
	ICPC-2	R96	3
Severe chronic pulmonary disease	ICD-10	J41–J44, J47, Z90.2	2
Type 2 diabetes requiring medication	ICD-10	E11, E13, E14	2,3
	ICPC-2	T90	3
Blood glucose lowering drugs, excluding insulin	ATC	A10B	4
Down syndrome	ICD-10	Q90	2, 3
**Predisposing to severe Covid-19**			
Severe heart disease	ICD-10	I11–I13, I15, I20–I25, I50	1, 2
Neurological illness or condition that affects breathing	ICD-10	G70-G73, G80–G83, I60–I69	2
Immunosuppressive drug therapy for autoimmune disease			
Autoimmune disease	ICD-10	D86, K50, K51, L40, M02, M05–M07, M13.9, M45, M46.0, M46.1, M46.9, M94.1	1, 2
Immunosuppressive drug therapy	ATC	H02AB02, H02AB04, H02AB06, H02AB07, L01BA01, L01XC02, L04AA06, L04AA10, L04AA13, L04AA18, L04AA24, L04AA26, L04AA29, L04AA33, L04AA37, L04AB, L04AC, L04AD01, L04AD02, L04AX01, L04AX03	4
Severe chronic liver disease	ICD-10	K70.2, K70.3, K70.4, K71–K74	2
Type 1 diabetes	ICD-10	E10	2, 3
	ICPC-2	T89	3
Insulin and analogues	ATC	A10A	4
Adrenal insufficiency	ICD-10	E25.0, E27.1, E27.2, E27.4, E31.00, E31.01, E31.08, E89.6	1, 2
Sleep apnea	ICD-10	G47.3	2, 3
Continuous positive airway pressure therapy	NCSP	WX723, WX780	2
Psychotic disorders	ICD-10	F20–F29	2, 3
	ICPC-2	P72	3
Clozapine	ATC	N05AH02	4

ATC, Anatomical Therapeutic Chemical Classification System; ICD-10, International Statistical Classification of Diseases and Related Health Problems, tenth revision; ICPC-2, International Classification of Primary Care, second edition; NCSP, Nordic Nomesco Classification of Surgical Procedures.

Registers: 1, Special Reimbursement Register for Medicine Expenses; 2, Care Register for Health Care; 3, Register of Primary Health Care Visits; 4, Prescription Centre database.
