# Supplementary material for: Effectiveness of vaccination against SARS-CoV-2 infection and Covid-19 hospitalisation among Finnish elderly and chronically ill—An interim analysis of a nationwide cohort study
Source: PLoS One. 2021 Nov 18;16(11):e0258704. doi: 10.1371/journal.pone.0258704 (PMC8601574; doi:10.1371/journal.pone.0258704)
Supplement: S2 Table — (PDF) [file pone.0258704.s002.pdf]

S2 Table: Distribution of baseline characteristics and percentage vaccinated first with mRNA or adenovirus vector (AdV) vaccine, Finnish elderly aged 70+ years.

	Number of study subjects	Percentage vaccinated first with mRNA vaccine	AdV vaccine
**Age in years**			
70-74	353956	79	10
75-79	231915	86	4
80-89	258389	89	1
90+	56832	83	1
**Sex**			
Male	384170	83	6
Female	516922	85	5
**Presence of medical conditions predisposing to severe Covid-19**			
No predisposing medical condition	376668	83	6
At least one highly predisposing medical condition	314949	85	5
At least one predisposing but no highly predisposing medical condition	209475	85	5
**In Helsinki-Uusimaa hospital district**			
No	685004	83	6
Yes	216088	87	2
**In long-term care**			
No	851206	84	5
Yes	49886	85	0
