# Supplementary material for: Effectiveness of vaccination against SARS-CoV-2 infection and Covid-19 hospitalisation among Finnish elderly and chronically ill—An interim analysis of a nationwide cohort study
Source: PLoS One. 2021 Nov 18;16(11):e0258704. doi: 10.1371/journal.pone.0258704 (PMC8601574; doi:10.1371/journal.pone.0258704)
Supplement: S3 Table — (PDF) [file pone.0258704.s003.pdf]

S3 Table: Distribution of baseline characteristics and percentage vaccinated first with mRNA or adenovirus vector (AdV) vaccine, chronically ill aged 16–69 years.

		Percentage vaccinated first with	
	Number of study subjects	mRNA vaccine	AdV vaccine
**Age in years**			
16-38	151887	33	4
39-51	151543	50	10
52-59	173280	58	19
60-64	138227	55	28
65-69	159589	32	54
**Sex**			
Male	403410	44	24
Female	371116	48	22
**Presence of medical conditions predisposing to severe Covid-19**			
At least one highly predisposing medical condition	329664	38	37
At least one predisposing but no highly predisposing medical condition	444862	51	12
**In Helsinki-Uusimaa hospital district**			
No	567944	46	22
Yes	206582	44	27
