# Supplementary material for: Effectiveness of vaccination against SARS-CoV-2 infection and Covid-19 hospitalisation among Finnish elderly and chronically ill—An interim analysis of a nationwide cohort study
Source: PLoS One. 2021 Nov 18;16(11):e0258704. doi: 10.1371/journal.pone.0258704 (PMC8601574; doi:10.1371/journal.pone.0258704)
Supplement: S4 Table — DSV, day since vaccination; Est., Point estimate; LCI, lower 95% confidence interval limit; UCI, upper 95% confidence interval limit. (PDF) [file pone.0258704.s004.pdf]

S4 Table: Crude and adjusted hazard ratios comparing the hazard of confirmed SARS-CoV-2 infection or Covid-19 hospitalization in study subjects who received exactly 1 or 2 doses of mRNA vaccine with the corresponding hazard in the unvaccinated, Finnish elderly aged 70+ years.

	**SARS-CoV-2 infection**						**Covid-19 hospitalization**					
	**Crude hazard ratio**			**Adjusted hazard ratio**			**Crude hazard ratio**			**Adjusted hazard ratio**		
	Est.	LCI	UCI	Est.	LCI	UCI	Est.	LCI	UCI	Est.	LCI	UCI
**First dose**												
0-6 DSV	0.773	0.624	0.956	0.669	0.541	0.827	0.465	0.270	0.801	0.425	0.246	0.733
7-13 DSV	0.846	0.684	1.046	0.707	0.572	0.875	0.644	0.400	1.038	0.577	0.357	0.931
14-20 DSV	0.766	0.607	0.966	0.618	0.490	0.780	0.831	0.538	1.285	0.734	0.473	1.138
21-27 DSV	0.745	0.581	0.956	0.589	0.459	0.755	0.498	0.286	0.869	0.433	0.248	0.758
28-34 DSV	0.693	0.527	0.911	0.534	0.406	0.701	0.470	0.263	0.841	0.414	0.231	0.742
35-41 DSV	0.735	0.554	0.976	0.545	0.411	0.722	0.411	0.218	0.774	0.360	0.191	0.680
42+ DSV	0.827	0.680	1.004	0.535	0.441	0.650	0.386	0.253	0.587	0.321	0.208	0.496
**Second dose**												
0-6 DSV	0.300	0.123	0.728	0.153	0.063	0.371	0.267	0.064	1.108	0.209	0.050	0.877
7+ DSV	0.725	0.530	0.990	0.250	0.181	0.347	0.087	0.021	0.355	0.072	0.017	0.300

DSV, day since vaccination; Est., Point estimate; LCI, lower 95% confidence interval limit; UCI, upper 95% confidence interval limit
