# Supplementary material for: Effectiveness of vaccination against SARS-CoV-2 infection and Covid-19 hospitalisation among Finnish elderly and chronically ill—An interim analysis of a nationwide cohort study
Source: PLoS One. 2021 Nov 18;16(11):e0258704. doi: 10.1371/journal.pone.0258704 (PMC8601574; doi:10.1371/journal.pone.0258704)
Supplement: S5 Table — DSV, day since vaccination; Est., Point estimate; LCI, lower 95% confidence interval limit; UCI, upper 95% confidence interval limit. (PDF) [file pone.0258704.s005.pdf]

S5 Table: Crude and adjusted hazard ratios comparing the hazard of confirmed SARS-CoV-2 infection or Covid-19 hospitalization in study subjects who received exactly 1 or 2 doses of mRNA vaccine with the corresponding hazard in the unvaccinated, chronically ill aged 16–69 years.

	**SARS-CoV-2 infection**						**Covid-19 hospitalization**					
	**Crude hazard ratio**			**Adjusted hazard ratio**			**Crude hazard ratio**			**Adjusted hazard ratio**		
	Est.	LCI	UCI	Est.	LCI	UCI	Est.	LCI	UCI	Est.	LCI	UCI
**First dose**												
0-6 DSV	0.568	0.438	0.737	0.612	0.472	0.794	0.142	0.035	0.574	0.129	0.032	0.521
7-13 DSV	0.605	0.462	0.792	0.664	0.506	0.870	0.242	0.077	0.763	0.218	0.069	0.687
14-20 DSV	0.519	0.380	0.708	0.575	0.421	0.785	0.569	0.249	1.304	0.511	0.223	1.171
21-27 DSV	0.532	0.378	0.749	0.587	0.417	0.827	0.120	0.017	0.862	0.106	0.015	0.764
28-34 DSV	0.392	0.251	0.614	0.424	0.271	0.664	0.162	0.022	1.168	0.139	0.019	1.008
35-41 DSV	0.386	0.222	0.670	0.411	0.236	0.714	Not estimated			Not estimated		
42+ DSV	0.686	0.506	0.928	0.857	0.632	1.161	0.411	0.131	1.287	0.410	0.131	1.288
**Second dose**												
0-6 DSV	0.115	0.016	0.817	0.127	0.018	0.906	Not estimated			Not estimated		
7+ DSV	0.217	0.139	0.336	0.227	0.146	0.353	0.095	0.013	0.678	0.100	0.014	0.710

DSV, day since vaccination; Est., Point estimate; LCI, lower 95% confidence interval limit; UCI, upper 95% confidence interval limit
