# Supplementary material for: Effectiveness of vaccination against SARS-CoV-2 infection and Covid-19 hospitalisation among Finnish elderly and chronically ill—An interim analysis of a nationwide cohort study
Source: PLoS One. 2021 Nov 18;16(11):e0258704. doi: 10.1371/journal.pone.0258704 (PMC8601574; doi:10.1371/journal.pone.0258704)
Supplement: S6 Table — DSV, days since vaccination; Est., Point estimate; LCI, lower 95% confidence interval limit; UCI, upper 95% confidence interval limit. (PDF) [file pone.0258704.s006.pdf]

S6 Table: Crude and adjusted hazard ratios comparing the hazard of confirmed SARS-CoV-2 infection or Covid-19 hospitalization in study subjects who received exactly 1 or 2 doses of adenovirus vector vaccine with the corresponding hazard in the unvaccinated, chronically ill aged 16–69 years.

	**SARS-CoV-2 infection**						**Covid-19 hospitalization**					
	**Crude hazard ratio**			**Adjusted hazard ratio**			**Crude hazard ratio**			**Adjusted hazard ratio**		
	Est.	LCI	UCI	Est.	LCI	UCI	Est.	LCI	UCI	Est.	LCI	UCI
**First dose**												
0-6 DSV	0.492	0.373	0.649	0.599	0.453	0.791	0.188	0.047	0.757	0.157	0.039	0.633
7-13 DSV	0.656	0.514	0.836	0.804	0.630	1.027	1.163	0.666	2.029	0.961	0.548	1.685
14-20 DSV	0.498	0.373	0.665	0.620	0.464	0.830	1.024	0.560	1.874	0.838	0.456	1.541
21-27 DSV	0.605	0.458	0.799	0.764	0.578	1.011	0.414	0.154	1.112	0.335	0.124	0.903
28-34 DSV	0.406	0.281	0.587	0.518	0.358	0.748	0.117	0.016	0.831	0.093	0.013	0.666
35-41 DSV	0.505	0.350	0.730	0.635	0.438	0.919	0.815	0.360	1.843	0.638	0.281	1.449
42+ DSV	0.401	0.318	0.507	0.497	0.393	0.630	0.564	0.327	0.973	0.423	0.243	0.736
**Second dose**												
0-6 DSV	0.687	0.220	2.147	1.375	0.439	4.309	Not estimated			Not estimated		
7+ DSV	Not estimated			Not estimated			Not estimated			Not estimated		

DSV, days since vaccination; Est., Point estimate; LCI, lower 95% confidence interval limit; UCI, upper 95% confidence interval limit
